# Effect of a 12-Week Rehabilitation Exercise Program on Shoulder Proprioception, Instability and Pain in Baseball Players with Shoulder Instability

**DOI:** 10.18502/ijph.v49i8.3890

**Published:** 2020-08

**Authors:** Jin-Ho YOON, Ki-Jae SONG, Mu-Yeop JI, Bang-Sub LEE, Jae-Keun OH

**Affiliations:** 1. Department of Sports Rehabilitation, Korea Nazarene University, Cheonan, Korea; 2. Department of Sports Medicine, Korea National Sport University, Seoul, Korea; 3. Department of Sports Science, Korea Institute of Sport Science, Chungcheongbok-do, Korea

**Keywords:** Baseball players, Proprioception, Rehabilitation exercise program, Shoulder instability, Visual analog scale

## Abstract

**Background:**

The shoulder joint has a wide range of motion, but is vulnerable to sport-related injuries. We aimed to evaluate the differences in the proprioception of the shoulder should instability, and shoulder pain in high school baseball players with shoulder instability following a 12-week rehabilitation exercise program.

**Methods:**

We enrolled 13 baseball players with shoulder instability who visited the Orthopedics Department at Konkook University Hospital in Seoul, South Korea and 12 controls without shoulder instability. We examined the dominant shoulder and the non-dominant shoulder for both groups. We restricted participation to individuals who had no other orthomechanical disease in the past six months, except for instability of the shoulder, and no physical limitation to participate in the exercise. We measured the proprioception of the shoulder and shoulder instability, and we also evaluated pain with the Visual Analog Scale before and after the rehabilitation program. To verify the differences between groups, we used a two-way analysis of variance, and a two-way analysis of covariance was used when a significant difference was found at the pretest (baseline between groups).

**Results:**

Proprioception was associated with shoulder instability. The Visual Analog Scale rating improved in the dominant shoulder with instability; and a positive change was found in the dominant shoulder without instability after the rehabilitation program (*P* < 0.05).

**Conclusion:**

The 12-week rehabilitation exercise program might improve the proprioception and pain of patients with shoulder instability. However, further studies with more participants and a rehabilitation exercise program should be undertaken.

## Introduction

The glenohumeral joint has the widest range of motion (ROM), and approximately 7% of all sports injuries involve a shoulder joint problem (1). The shoulder consists of three bones; the clavicle, scapula, and humerus, and four joints, which are the sternoclavicular, acromioclavicular, glenohumeral (GH), and scapulothoracic joints (2). The GH joint, commonly known as the shoulder joint, provides stability and maximum range of motion to various structures such as the muscles, ligaments, and tendons, and also includes a group of muscles and tendons called the rotator cuff. The GH joint enables the various motions required for sports activities, but it also increases the risk of injury (2). The GH joint is a ball-socket joint that consists of a humeral head attached to the glenoid fossa; the humeral head is surrounded by ligaments and muscles for stability (3). Thus, damage to the shoulder joint stabilizing structures can induce a shoulder injury (4–5).

Proprioception, also known as kinesthesia, is the sense of limb movement and position that was originally described by Sherrington in 1907. It encompasses joint position sense, which is the ability to identify the location of the arms and legs (6–7). Proprioception involves neural signaling to the central nervous system through the Golgi tendon organs and muscle spindles that are located in various tissues, including the skin, muscles, ligaments, tendons, and the articular capsule. A decline of proprioception regulative function can induce functional instability in joint movement (8–9). Muscular fatigue reduces the joint position sense, which leads to a high risk of injury (10). A study that measured proprioception in 20 over-head throwing baseball players showed a decline of function of the dominant shoulder compared to the non-dominant shoulder. They reported that muscular fatigue due to the throwing motion lowered proprioception ability, and caused an accumulation of microtrauma and instability (11). However, it is not clear whether the shoulder instability in these players is due to a change in proprioception caused by sports activities or a decline in proprioception caused by over-use damage that is linked to a more severe alteration of the stabilizing structures (12). Thus, the change in the ROM and proprioception in over-head throwing baseball players can result from the structural change of the shoulder joint that occurs as a physiological adaptation to the throwing characteristics.

Pain is usually expressed as an unpleasant feeling based on the threshold level that was registered by cognitive memory. It arises from the irritation of the pain receptors by a noxious stimulus, triggering the relay of an electrical signal to the central nervous system (13–14). Pain is one of the most representative and observable symptoms of musculoskeletal disease, but it is hard to measure because it is a subjective experience that is different for each person (15). Thus, the Visual Analog Scale (VAS) was developed to aid in obtaining the overall pain degree index (16). The strength of this measurement tool lies in the simple instructions and the ease of grading because it measures the degree of pain by the length from the initial point to the marked point; thus, it is well adapted to clinical practice (17–19).

Recently, many researchers have expressed interest in determining the effect of the structural adaptation on the stability of the GH joint, and its association with injury (20). The anatomical change in the GH joint following exposure to chronic stress can be regarded as an adaptation to exercise participation, and requires a change in the stability structures to ensure shoulder joint stability. In this regard, the purpose of exercise rehabilitation for instability of the GH joint should be the prevention of shoulder damage and inhibition of recurring injury. Rehabilitation should focus on the reinforcement of the dynamic muscular function of the soft tissue that contributes to GH joint stability and improvement of proprioception (21–22).

Therefore, we investigated the change of the GH joint proprioception ability of high school baseball players with shoulder instability who were enrolled in a 12-week rehabilitative training program, and provide research data for exercise rehabilitation.

## Methods

### Participants

Our study included high school baseball players who visited the Orthopedics Department at Konkook University Hospital in Seoul, South Korea and were diagnosed with shoulder instability.

All study participants provided informed consent, and the study design was approved by the Korea National Sports University.

Based on the power analysis using the G-Power program (G* Power 3.1.7, Heinrich-Heine-University, Düsseldorf, Germany), a sample size of 24 participants was required to detect an effect size of 0.05 with type 1 error of 0.05 and statistical power of 0.95.

The inclusion criteria were the lack of history of orthopedic disease in the past six months except for instability of the shoulder, and the absence of any restriction to participate in the exercise. We excluded individuals who had received any medication or treatment that could affect the study results. Considering the possibility of drop-outs, we included a total of 25 male participants who were evenly assigned to the patient (P) (n = 12) and healthy (H) (n = 13) groups. We excluded one participant who did not complete the 12-week exercise rehabilitation therapy. Table 1 shows the characteristics of the participants in cluded in this study.

**Table 1:** Characteristics of the participants

***Variables***	***Patient (n = 12)***	***Healthy (n = 13)***	***P***
Age (yr)	17.82 ± 1.40	17.57 ± 1.87	0.723
Height (cm)	178.45 ± 4.03	179.93 ± 7.10	0.554
Weight (kg)	84.76 ± 9.34	82.21 ± 8.41	0.744
Body mass index (kg/m^2^)	26.64 ± 3.03	25.39 ± 2.17	0.377
Percent body fat (%)	20.86 ± 5.97	17.83 ± 3.27	0.285

Values are means ± standard deviations.

Statistical significance is evaluated by an independent *t*-test

### Measurement of proprioception ability in the shoulder joint

We referred to the dominantly used shoulder as dominant and the opposite shoulder as non-dominant. The GH joint proprioception test encompasses the threshold that detects passive movement and joint position sense that regenerates a given active angle. In this study, we used active joint position sense measurements to assess the proprioception ability of the shoulder joint. We measured the internal and external rotation proprioception using the isokinetic equipment HUMAC NORM (Stoughton, MA, USA). We used the active method in which every participant was expected to move the joint to achieve the target angle We set the starting angle at 0, and the target angle was calculated by subtracting 10% from the ROM of the internal and external rotation after calculating 10% of the summed ROM of the internal and external rotations (12). After covering the participant’s eyes, the examiner instructed the participant to reach the predetermined target angle. Ten seconds later, the participant attempted to reproduce the memorized angle from the starting angle. The maximum error value from the target angle was excluded, and the mean of 2 measurements were calculated for use in this study.

### Visual analog scale

We used the VAS to quantify and measure the subjective degree of pain experienced by the participants. The VAS is one of the most commonly used methods to measure the degree of pain, which is scored as no pain, a little pain, normal pain, and severe pain based on a horizontal millimeter-scale of 0–4 mm, 5–44 mm, 45–74 mm, and 75–100 mm, respectively (23). All participants received education before the measurement of the VAS ratings. The VAS ratings were measured before and after the experiment.

### Rehabilitation exercise training

The rehabilitation exercise training was a four-stage program (24). The four stages include the acute phase (phase one), intermediate phase (phase two), advanced strengthening phase (phase three), and return-to-activity phase (phase four) (24). In consultation with two experts who had over ten years of experience in rehabilitative training, we reorganized some activities in each of the four phases to account for the participants who were still receiving hospital care. We held two meetings with the experts to constitute a relevant program that was then applied identically to both the injured and healthy shoulders. We monitored the participants through the four stages of the program via observation and interviews. Details of the rehabilitation exercise training are shown in Table 2.

**Table 2:** The rehabilitation exercise training protocol

***Stage***	***Goal***	***Exercises and modalities***
(I) Acute phase	(1) Diminish pain and inflammation (2) Normalize motion (3) Retard muscular atrophy (4) Reestablish dynamic stability (muscular balance) (5) Control functional stress/strain	Cryotherapy, ultrasound, electrical stimulation Flexibility and stretching for posterior shoulder muscles (improve internal rotation and horizontal adduction) Rotator cuff strengthening (especially external rotator muscles) Scapular muscle strengthening (especially retractor, protractor, depressor muscles) Dynamic stabilization exercises (rhythmic stabilization) Closed kinetic chain exercises Proprioception training Abstain from throwing
(II) Intermediate phase	(1) Progress strengthening exercise (2) Restore muscular balance (external/internal rotation) (3) Enhance dynamic stability (4) Control flexibility and stretches 4 – 8 weeks	Continue stretching and flexibility (especially internal rotation and horizontal adduction) Progress isotonic strengthening Complete shoulder program Thrower’s Ten program Rhythmic stabilization drills Initiate core strengthening program Initiate leg program
(III) Advanced strengthening phase	(1) Aggressive strengthening (2) Progress neuromuscular control (3) Improve strength, power, and endurance (4) Initiate light throwing activities	Flexibility and stretching Rhythmic stabilization drills Thrower’s Ten program Initiate plyometric program Initiate endurance drills Initiate short-distance throwing program
(IV) Return to activity phase	(1) Progress to throwing program (2) Return to competitive throwing (3) Continue strengthening and flexibility drills	Stretching and flexibility drills Thrower’s Ten program Plyometric program Progress interval throwing program to competitive Throwing

### Statistical analysis

We expressed all values as means ± standard deviations by using Windows SPSS version 18.0 (IBM Corp., Armonk, NY, USA). We conducted a one-way analysis of covariance (post value) to determine the effect of the exercise program on proprioception of the shoulder joint with the pre-intervention group difference as the covariate. For the post-hoc test, we conducted a paired or independent *t*-test to determine the pre-intervention and post-intervention differences and intergroup variations. We set the statistical significance at *P* < 0.05.

## Results

The internal rotation proprioception before rehabilitation had a significant effect on the proprioception after rehabilitation (*P*=0.043). The analysis of pre- and post-intervention differences of internal rotation proprioception showed a 55.92% decrease for the patient-dominant shoulder joint (pre 10.63 ± 4.06°, and post 4.68 ± 3.16°; *P*< 0.001) and a 24.03% decrease for the patient-non-dominant shoulder joint (pre 7.08 ± 3.75°, and post 5.38 ± 5.07°; *P* = 0.047). The healthy-dominant shoulder joint showed a 56.79% decrease of internal rotation proprioception (pre 7.53 ± 4.04°, and post 3.25 ± 1.74°; *P* < 0.001) (Table 3).

**Table 3:** Changes in internal rotation proprioception between groups

***Variable***	***Group***	***Pre***	***Post***	***F***	***p***
Internal rotation proprioception (°)	Patient dominant shoulder	10.63 ± 4.06	4.68 ± 3.16^***^	3.031	0.043
	Patient non-dominant shoulder	7.08 ± 3.75^+^	5.38 ± 5.07^#^		
	Healthy dominant shoulder	7.53 ± 4.04	3.25 ± 1.74^∫∫∫^		

Values are means ± standard deviations

Tested by one-way analysis of covariance (post value) adjusted pre-value, and a paired or an independent *t*-test

*** *P* < 0.001, patient dominant shoulder, pre vs. post

# *P* < 0.05, healthy dominant shoulder, pre vs. post

∫∫∫ *P* < 0.001, healthy dominant shoulder, pre vs. post

+ *P* < 0.05, patient dominant shoulder, pre vs. patient non-dominant pre Pre, before rehabilitation training; Post, after rehabilitation training

The external rotation proprioception before rehabilitation had a significant effect on the proprioception after rehabilitation (*P*= 0.003). The analysis of pre- and post-intervention differences in external rotation proprioception showed a 55.13% decrease in the patient-dominant shoulder joint (pre 8.86 ± 2.88°, and post 3.98 ± 2.32°; *P*<0.001) and a 32.21% decrease in the patient-non-dominant shoulder joint (pre 7.63 ± 3.53°, and post 5.18 ± 2.74°; *P*=0.016). The healthy-dominant shoulder joint showed a 23.69% decrease in external rotation proprioception (pre 5.88 ± 2.39°, and post 4.48 ± 2.60°; *P*<0.037) (Table 4).

**Table 4:** Changes in external rotation proprioception between groups

***Variable***	***Group***	***Pre***	***Post***	***F***	***P***
External rotation proprioception (°)	Patient dominant shoulder	8.86 ± 2.88	3.98 ± 2.32^***^	5.679	0.003
	Patient non-dominant shoulder	7.63 ± 3.35	5.18 ± 2.74^#^		
	Healthy dominant shoulder	5.88 ± 2.39^+^	4.48 ± 2.60^∫^		

Values are means ± standard deviations

Tested by one-way analysis of covariance (post value) adjusted pre-value and a paired or an independent *t*-test

*** *P* < 0.001, patient dominant shoulder pre vs. post

# *P* < 0.05, patient non-dominant shoulder pre vs. post

∫ *P* < 0.05, healthy dominant shoulder pre vs. post

+ *P* < 0.05, healthy dominant shoulder pre vs. patient dominant shoulder pre Pre, before rehabilitation training; Post, after rehabilitation training

We noticed a significant decrease in the patient group with shoulder joint instability after rehabilitation (pre 36.67 ± 10.73 mm, and post 10.00 ± 6.03 mm, *P*<0.001). Similarly, the healthy group registered a significant decrease in the shoulder joint instability (pre 6.15 ± 7.68 mm, and post 0.00 ± 0.00 mm; *P*=0.014) (Table 5).

**Table 5:** Changes in the Visual Analog Scale between groups

***Variable***	***Group***	***Pre***	***Post***	***F***	***P***
Visual analog scale (mm)	Patient dominant shoulder	36.67 ± 10.7	10.00 ± 6.03^***^	17.160	< 0.001
	Healthy dominant shoulder	6.15 ± 7.68^+++^	0.00 ± 0.00^#^		

Values are means ± standard deviations

Tested by one-way analysis of covariance (post value) adjusted pre-value and a paired or an independent *t*-test

*** *p <* 0.001, patient dominant shoulder pre vs. post

# *p* < 0.05, healthy dominant shoulder pre vs. post

+++ *P <* 0.001, healthy dominant shoulder pre vs. patient dominant shoulder pre Pre, before rehabilitation training; Post, after rehabilitation training

## Discussion

We noted significant improvement in proprioception and degree of pain of the shoulder joint of baseball players following the 12-week rehabilitation exercise.

Conscious proprioception is necessary for proper joint function in sports, occupation, and daily life, while unconscious proprioception regulates muscular function for stability (25–26). The labrum and the articular capsule-ligament were necessary for control of the location and movement of the GH joint (27). Lowered proprioception is closely related to a decrease of motor function in athletes with a sports injury, which suggested that recovery of proprioception through the whole rehabilitation process was an important indicator of the degree of recovery (6,9).

In this study, the proprioception of the dominant shoulder with instability after the 12-week rehabilitation was significantly lower compared with the proprioception of the non-dominant shoulder, and the healthy dominant shoulder during internal rotation and external rotation, respectively. This is consistent with other studies, where instability of the shoulder joint had a negative effect on its proprioception sense, and suggested that the improvement of shoulder proprioception was necessary for the recovery of shoulder instability (28–29). Moreover, the improvement of proprioception at the internal and external rotations of the dominant shoulder with instability following the rehabilitation exercise suggests that the exercise program is essential for proprioceptive neuromuscular facilitation that enhances the proprioception sense. Moreover, significant improvement of the internal rotation proprioception of the dominant shoulder was found in healthy players (without shoulder instability), and a positive change of external rotation proprioception was observed, though it was not statistically significant. Notably, the positive change of proprioception of both internal and external rotation after the rehabilitative exercise suggests that the rehabilitative exercise program is effective for the improvement of shoulder proprioception regardless of shoulder instability. The changes in proprioception represented an improvement in the shoulder joint ROM. An increased degree of extension in players with repetitive throwing motion had an adverse effect on proprioception (30).

The utility of VAS to measure the effectiveness of rehabilitative training to address exercise-related musculoskeletal injury is increasing (31–32). For instance, Hübscher et al., who studied the painkilling effect of acupuncture on delayed-onset muscle soreness (DOMS) after exercise, reported that the VAS rating was decreased in the treatment group even though evidence of muscular function and the mechanism of pain relief were not elucidated (33). Mori et al., who investigated the effect of massage on pain after spine isometric exercise, reported an improvement of the VAS in the massage group compared to the control group, but the evidence for pain relief was not found (34).

These studies used the VAS for a comprehensive interpretation of pain with minimal consideration for the specific pain-related quality. The VAS only measures the intensity of pain; thus, it may be considered a one-dimensional tool for pain assessment despite the high relative sensitivity, distribution of response, preference, and reliability. This attribute implies that the VAS only measured a degree of the unpleasant feeling of the responder rather than the direct consideration of the cause of the pain. Notably, pain is not just sensory, but is also a sum of sensation and emotion; therefore, it can be regarded as a perception rather than a sensation (35).

Pain in individuals with shoulder instability arises from various causes. In this study, the group without shoulder instability also showed slight pain before rehabilitation, suggesting a perception of discomfort regardless of instability. Even though the question about pain degree was restricted to the shoulder, the reported pain level could be the sum of the overall body discomfort. Cognition of the overall body affects the degree of pain (24–26). In this regard, the rehabilitation exercise program has a positive effect to relieve the overall body discomfort, including the shoulder, because both the patients and healthy participants showed a positive change of pain after participating in the rehabilitation exercise.

The study included the following limitations that affect the validity of the findings. First, sampling was limited to baseball players even though there were many sports events that require overhead throwing motion, such as volleyball and basket-ball. Future studies should recruit participants from different sports fields. Second, the male participants were recruited from a hospital in Seoul; consequently, the findings could not be generalized to all Korean baseball players. Third, even though the required number of participants was verified using the G-Power program, only a few individuals (n = 25) participated in this study. However, the small sample size is justifiable since it is difficult to recruit a patient population, especially for a 12-week rehabilitation exercise program. Nevertheless, future studies should recruit more participants to improve the study rigor.

## Conclusion

The 12-week rehabilitation exercise program evaluated in this study has proven effective for the improvement of proprioception and pain in patients with shoulder instability. However, the findings should be confirmed using a larger study that evaluates a longer rehabilitation exercise program.

## Ethical considerations

Ethical issues (Including plagiarism, informed consent, misconduct, data fabrication and/or falsification, double publication and/or submission, redundancy, etc.) have been completely observed by the authors.
